# Age‐mediation of tree‐growth responses to experimental warming in the northeastern Tibetan Plateau

**DOI:** 10.1002/ece3.4920

**Published:** 2019-01-28

**Authors:** Jun Du, Kai Li, Zhibin He, Longfei Chen, Xi Zhu, Pengfei Lin

**Affiliations:** ^1^ Linze Inland River Basin Research Station Chinese Ecosystem Research Network Lanzhou China; ^2^ Key Laboratory of Ecohydrology of Inland River Basin, Northwest Institute of Eco‐Environment and Resources Chinese Academy of Sciences Lanzhou China; ^3^ Key Laboratory of Western China's Environmental Systems, College of Earth Environmental Sciences Lanzhou University Lanzhou China; ^4^ Department of Agricultural and Applied Economics Texas Tech University Lubbock Texas; ^5^ University of Chinese Academy of Sciences Beijing China

**Keywords:** age mediation, experimental warming, phenology, sapling growth, Tibetan Plateau

## Abstract

The trajectory of tree‐growth response to climate warming may be related to attributes like tree age. However, age‐mediation of temperature sensitivity of tree growth has received little attention. This study aimed to determine how age affects tree growth in a future warmer world. In a 2‐year ecosystem warming experiment in the northeastern Tibetan Plateau of China, we explored the response of Qinghai spruce saplings at two life stages to two warming levels. Our results indicated a significant interaction between warming and age for sapling growth of Qinghai spruce. In high‐level warming scenario, the experiment increased growing season air temperatures by approximately 1.0°C and annual growing degree‐days by 38%. In response, warmed saplings lengthened the growing season by 10 days on average and increased the final shoot length to a maximum of 104% compared to control groups. Comparison of age classes revealed that old saplings exhibited significantly higher temperature sensitivity than young saplings. This performance may be caused by the differences in adaptive strategy to the asymmetric warming occurring during the whole day. Increased daytime temperature was expected to significantly enhance leaf photosynthesis, whereas lack of obvious nighttime warming would effectively restrict autotrophic respiration, thus resulting in the higher growth rate of old saplings compared with young saplings. Moreover, lack of nighttime warming rendered young saplings to be still in high stresses of freezing injury at low temperatures. These findings highlight the need for additional research on the effects of further climate anomalies on tree species during their ontogenetic processes.

## 1 INTRODUCTION

The Tibetan Plateau (TP) experienced a temperature rise of 0.4°C per decade during the past 50 years, twice the global rate of increase (Dong, Jiang, Zheng, & Zhang, 2012). Regional climate projections indicated a continuing trend toward warmer conditions throughout the TP, with increases of 1.8–4.1°C by the end of this century (Su, Duan, Chen, Hao, & Cuo, 2013). Significant warming trends inevitably result in consequences for plant systems in terms of plant demography, community composition, and population dynamics (Danby & Hik, 2007; Lenoir, Gégout, Marquet, Ruffray, & Brisse, 2008; Ruiz‐Labourdette, Schmitz, & Pineda, 2013; Sivadasan et al., 2017; Zhou et al., 2016). Documented changes in the phenology and reproduction in the cold forest biome in the TP, for example, are among the most evident effects of climate change (Piao, Fang, & He, 2006; Zhang, Zhang, Dong, & Xiao, 2013). The potentially prolonged photosynthetic season may benefit net carbon uptake into forest as well as the climate‐feedback functioning (He et al., 2015). Indeed, this trajectory can be complex, because tree performance in response to climate warming may be mediated by traits related to resource acquisition (Chen, An, Inouye, & Schwartz, 2015; Dorji et al., 2013; Estiarte & Peñuelas, 2015; Schwartz, Hanes, & Liang, 2014), such as life‐history phases (juvenile vs. mature forest). However, variability in phenological plasticity and sensitivity to elevated temperature over the plant life cycle is not well known; we lack the insight on how age structure affects tree growth in the future warmer world (Esper, Niederer, Bebi, & Frank, 2008; Ruiz‐Benito et al., 2015; Xu, Yin, Xiong, Wan, & Liu, 2012).

Previous studies indicated that different plant species in diverse life histories may respond differently to climate warming (Baldocchi, Xu, & Kiang, 2004; Dorji et al., 2013; Rollinson & Kaye, 2012). Early‐flowering species, for example, are often more vulnerable to climate anomalies than late‐flowering ones (Polgar & Primack, 2011). Even within a species, temperature sensitivity of phenology and growth can vary in different life‐cycle stages (Vitasse, 2013). For woody plants, ecophysiological traits such as metabolic rate, carbon assimilation, and water use efficiency generally differ with tree age, resulting in variable plasticity and adaptability to changing conditions (Mediavilla & Escudero, 2003; Xu et al., 2012). As a result of different physiological adaptations, climate change presumably leads to divergent strategies under different selective pressures depending on the growth phase (Mediavilla & Escudero, 2003). Thus, there is quite need to concern about age effects in researches on response to climate change.

To date, comparative studies have investigated on phenology patterns of young and mature growth as well as the environmental sensitivities (Esper et al., 2008; Li et al., 2013; Ruiz‐Benito et al., 2015). Compared to adult trees of deciduous species, seeding and saplings have been found to be characterized by an earlier flushing, an earlier xylem formation, a longer growth season and a growth rate (Augspurger & Bartlett, 2003; Li et al., 2013; Vitasse, 2013). Although investigations of the phenological difference of seedling and adult life stages are relevant to assess the impact of climate change on the fitness and growth of tree populations, it is still pressing to investigate the responses of premature growth for different age classes to climate change, since the forest age structure in China is dominated by young and mid‐aged types (Guo, Fang, Pan, & Birdsey, 2010). The importance of early stages of tree development in enabling successful establishment in new locations has been generally accepted (Danby & Hik, 2007; Kaye & Wagner, 2014). This launched a strong motivation for estimating tree performance at different phases of juvenile growth in response to episodes of warm temperatures. Knowledge of temperature sensitivity of phenology and growth of different age groups during the early tree life stages may clarify the status of interspecific competition and inform alternative management practices.

In this study, we aimed to investigate age‐mediation of a juvenile forest response to experimental warming. Our primary research question was whether saplings of different ages would exhibit divergent performances when exposed to different warming conditions. Current efforts to assess potential effects of climate change on species often include manipulation experiments (Rollinson & Kaye, 2012). A considerable number of field‐based warming studies involved a single plant or a small population, while warming at ecosystem level has been scarce, especially with different aged plants (Bronson, Gower, Tanner, & Van Herk, 2009; Danby & Hik, 2007; Fu, Campioli, Deckmyn, & Janssens, 2013; Kaye & Wagner, 2014; Xu et al., 2012). Seedlings and saplings in natural regenerated forests often display aggregate rather than uniform distribution before self‐thinning (He, Zhao, Liu, & Zhang, 2010), thereby warming in natural juvenile populations may be necessary at ecosystem level. In this study, an ecosystem warming experiment was designed in two‐aged sapling populations of Qinghai spruce (*Picea crassifolia*) at the lower elevation limits, where greatest warming‐related stress would be expected. *P. crassifolia*, a secondary conifer, is the dominant species in the northeastern Tibetan Plateau and is vulnerable to the impacts of climate change (Du, He, Yang, Chen, & Zhu, 2014). The low‐elevation forests have remained relatively understudied compared to the high‐elevation populations despite widespread tree mortality due to drought stress and intense competition (Wagner et al., 2015). Forest recruitment determines to a large extent how the distribution shifts in such a marginal environment.

Based on the results of previous studies of deciduous tree species (i.e., juvenile trees had a higher environmental sensitivity than mature trees; Li et al., 2013; Ruiz‐Benito et al., 2015), we expected that younger spruce saplings would exhibit an earlier spring phenology, a longer growing season, and higher growth rates than older saplings. Moreover, as a result of the ecophysiological adaptive mechanism to thermal stress, we also hypothesized that a small change in temperature would be sufficient to increase variability in sapling growth performance. Results of this study will permit more accurate modeling of forest growth and C sequestration in a future warmer world.

## 2 MATERIALS AND METHODS

### 2.1 Site description

The study was conducted at the lower elevational treeline on the north flank of the Qilian Mountains in the northeastern Tibetan Plateau, China (38.55^o^N, 100.28^o^E), at approximately 2,700 m elevation. Forests of pure Qinghai spruce (*P. crassifolia*) in this region have the upper limits of distribution at about 3,800 m above sea level (a.s.l.). They are naturally regenerated following widespread logging around the middle of the 20th century and targeted for conservation since 1988 (Du et al., 2014). The average density of timbers is about 2,400 stems/ha, and the leaf area index (LAI) is approximately 2.0 (Du et al., 2014). In most cases, field identification of juvenile populations belonging to the same age group was not difficult probably due to explosive germination in a favorable time, characterized by the roughly uniform heights and basal diameters in the same aggregate (He et al., 2010). In general, the understory is poorly developed, with moss and sparse shrubs (e.g., *Potentilla fruticosa* and *Caragana jubata*) and herbs (e.g., *Polygonum viviparum* and *Stipa ssp*) in the forest floor (He et al., 2015). Soils are aridic but generally well drained Haplustalfs, with the main parent material being calcareous rock. According to observational records from the meteorological station in the study site (38^o^33′22″N, 100^o^17′6″E, 2,750 m a.s.l.), the mean annual temperature is 2.65°C (for years 2000–2015), and annual cumulative precipitation is 386 mm. About 90% of precipitation concentrates as rainfall in May to September, resulting in low snowfall in winter (mean winter snow depth <5 mm, Peng, Piao, Ciais, Fang, & Wang, 2010). In the Qilian Mountains, the daily mean temperature is commonly above 0 starting at the end of March, and snow cover melts over a short period of time due to the high insulation (Dorji et al., 2013). The growth of spruce generally commences in May, and lasts until September (Tian et al., 2017), and is thought to be relatively insensitive to snow cover due to the large time lags (more than one month) between snowmelt and the onset of growth.

### 2.2 Experimental design

The experimental field is located on the north‐facing slope of approximately 20^o^. Eight 6.0 × 10.0 m open‐top chambers (OTCs) grouped into four blocks were constructed in September 2013 around naturally regenerated saplings in two life‐cycle stages, named hereafter young (average age = 17 years) and old (average age = 28 years; Table 1 & Supporting Information Figure S1). These two life‐cycle stages were representative of age groups recognized by the State Forestry Administration (Guo et al., 2010). In China, age classes of spruce forests are generally classified according to the principle of a grade of every ten years (State Forestry Administration of China, 2014). The age of each sapling was determined using annual ring counts and the number of annual nodes (He et al., 2010). These blocks were set on four large natural populations. Monitoring confirmed the relative consistency in micrometeorological conditions among control groups of the blocks (Supporting Information Table S1). Moreover, soil sampling by Chen, He, Du, Yang, and Zhu (2016) also revealed a non‐significant difference in soil depth, nutrient status, and light condition of each block site. The OTCs were made of polycarbonate with 90% of sunlight penetration. To minimize the shading and transmittance effects of chambers, a 1.0‐m buffer strip within each chamber was reserved at the perimeter, and an average of 30 saplings was enclosed inside (Table 1). Before we started the experiment, saplings that were significantly different in age than the average in each targeted group were removed to normalize the population age.

**Table 1:** The characteristics of sapling populations in each block

	*D*(genets)	DBH (cm)	*A* (year)	*H*(m)
Block 1	30 (5)a	3.4 (0.3)a	28 (4.6)a	1.8 (0.4)a
Block 2	37 (6)a	2.8 (0.2)b	18 (2.8)b	1.2 (0.3)b
Block 3	27 (6)a	3.3 (0.4)a	27 (3.0)a	1.9 (0.3)a
Block 4	33 (4)a	2.7 (0.3)b	16 (3.0)b	1.1 (0.2)b

Notes

Shown are mean values with standard deviation in parentheses. Different letters among blocks indicate significant differences in the characteristics of sapling populations (the Bonferroni's test)

*A*: averaged population ages; *D*: density of saplings; DBH: diameter at basal height (30 cm above the ground); *H*: mean height of saplings.

Two warming scenarios were included in the experimental design of this study based on global climate predictions as follows: first, a temperature increase of 0.5–1.0°C over the next two decades and second, an increase of 1.0–2.0°C late in this century (Su et al., 2013). To achieve the intended warming effects, OTCs extensively used in warming studies were improved in our design. Compared with the prior passive OTCs, our design was unique in that we were able to alter the intensity of thermal convection inside the OTCs by regulating the height of the chambers. Specifically, the distinct warming levels were realized by setting the height of the chambers to 2.0 m or 4.0 m, designated as “T+” and “T++”, respectively, where 2.0‐m height and T+ corresponded to the first warming scenario, and 4.0 m height and T++ corresponded to the second scenario. The paired chambers in each block were randomly assigned to the two treatments. “C” was used for the control groups. Therefore, our experiment contained the following treatments: old saplings + “T+”, old saplings + “T++”, young saplings + “T+”, young saplings + “T++” in a two‐factorial randomized complete block design (Supporting Information Figure S1).

### 2.3 Meteorological measurements

Air temperature and humidity were monitored within and outside the chambers every half‐hour at 1.5 m above ground using the EasyLog data loggers (DATAQ Instruments Inc., Akron, OH, USA). Soil temperature and moisture were recorded continuously using ECH2O‐5TM probes (Decagon Inc., Pullman, WA, USA) at 10 and 40 cm depth; these measurements were averaged for each half‐hour. Warming performance was assessed by comparing measurements between the warmed chambers and the control groups. All meteorological sensors were located near the center of the chambers whenever possible and installed more than 1 month before chamber construction (since July, 2013) to determine whether any differences were present in background values among plant populations. Eventually, no population effect was revealed, given that non‐significant differences were detected in micrometeorological conditions between control groups of the blocks (Supporting Information Table S1). Ambient precipitation was measured with a tipping‐bucket rain gauge.

### 2.4 Phenology and growth

We divided the chambers into ten 2.0 × 3.0 m cells; then, two saplings within each cell were randomly selected for measurements, for a total of 20 individuals per chamber. For chambers in block 1 and block 3, there were some saplings (generally no more than 10% in total) that were a little taller than the height of chambers; accordingly, these individuals were not considered when measurements were made. For the control group in each block, the determination of sampling trees was similar to that of warming treatments. Primary measurements in the study included spring phenology and shoot growth. Bud phenological stages were observed every other day during the growing seasons (May to September) of 2014 and 2015. Monitoring in spring was strengthened one day at a time. We recorded the dates of the following four stages (Huang, Deslauriers, & Rossi, 2014; Vitasse, 2013): (a) bud swelling, characterized by smooth bud scales with no visible needles; (b) bud burst, when the needles became apparent but still tightly clustered; (c) the onset of shoot growth when the needles fully expanded, and (d) the end of shoot growth, when the growth rate was <1 mm/day. The shoot‐growing season was then calculated as the number of days between the date of growth cessation (stage 4) and commencement (stage 3).

Sapling shoot growth was quantified by measuring the annual increment of vertical, lateral, and radial growth in late fall. Each selected sapling had only one vertical leader. The annual increment of vertical growth was determined by the shoot length from the most recent bud scar to that formed in the year prior (Danby & Hik, 2007). Monitoring for lateral branching included the uppermost branches with a minimum of one‐year‐old growth and the lower branches with a maximum of 3‐year growth. The measurement of lateral growth was similar to the measurement of the vertical leader. In the spring of 2014 and 2015, new shoot growth was measured with vernier calipers to the nearest 0.1 mm every other day beginning in May and until the end of July. Given the S‐shaped pattern of shoot elongation, the growth was fitted by a Gompertz function, widely applied to simulate growth of plant populations and to reduce non‐systematic errors due to manual measurements (Huang et al., 2014). The growth rate was then obtained from the first derivative of the function. We calculated the maximum and the average growth rates of vertical and lateral branches during the growing season. The yearly total length of branches (including lateral branch and vertical leader) was measured for four consecutive years (2012–2015), including two years before the study (2012 and 2013). At the end of September in 2015, we also determined the annual ring widths for 2012 through 2015 with a microscope for stem sections of ten randomly selected saplings in each chamber and control group. In addition to absolute growth, we calculated the relative growth of individuals during the course of the experiment (2014 and 2015). The relative increments of shoot length (RGI, relative growth index) were calculated as (value)/(mean of 2012 and 2013 values) to correct for inherent growth differences among individuals; the denominator was the average shoot growth value before the start of the experiment.

### 2.5 Statistical analysis

Meteorological datasets for continuous monitoring were divided into the growing season (May to September) and non‐growing season (October to April). A one‐way analysis of variance (ANOVA) was performed to compare the micrometeorological conditions during the growing season among control groups of the blocks. The performance of the OTCs was tested by comparing measurements between chambers and control group in each block. Warming efficacy during the two periods (growing season and non‐growing season) was assessed, respectively. We also quantified the accumulated thermal time for each treatment by calculating growing degree‐days (GDDs) with the equation GGD = [(*T*_max_ + *T*_min_)/2] − *T*_base_ with *T*_base_ = 5°C (Kaye & Wagner, 2014). Cumulative GGD was calculated for 2014 and 2015 starting from Julian Day 1 of that year to the end of September. Given that the OTC experiment might cause asymmetric warming occurring during the whole day (e.g., Danby & Hik, 2007), we compared the daytime (6:00 a.m. to 18:00 p.m.) temperature changes with that of nighttime (18:00 p.m. to 6:00 a.m.).

A one‐way ANOVA was also used to compare the population characteristics (including density of population, height of sapling, sapling age, and diameter at basal height) among blocks as well as sapling phenology and growth. ANOVAs revealed that there was no significant difference in population characteristics and sapling growth for both blocks in each age group (all at *p* > 0.1, Table 1 & Supporting Information Table S2). We therefore pooled the data for both blocks per age group for subsequent analysis and graphs. When comparing the sapling growth in warmed chambers and in control groups, paired *t* tests were used to evaluate the treatment effects. Sensitivities of phenology and growth to experimental warming were further assessed by defining values as ${\text{(Phenology}}_{\text{warming}}-{\text{Phenology}}_{\bar{\text{control}}})$ and ${\text{(RGI}}_{\text{warming}}-{\text{RGI}}_{\bar{\text{control}}})$ where ${\text{Phenology}}_{\bar{\text{control}}}/{\text{RGI}}_{\bar{\text{control}}}$ represented the corresponding averaged measurements of controlled manipulations. Warming and age effects on phenology and shoot growth were analyzed using two‐way repeated‐measures ANOVAs with the individual as a random effect, year as the repeated factor, and Julian day of phenological event or growth rate/relative increment of shoot growth as response variables. Before analysis, all data were checked for homogeneity of variances and normality. A Bonferroni's test was used for multiple comparisons of treatment and age effects. Significance was assigned at alpha = 0.05. All analyses were performed with the SAS 9.2 software (SAS Institute Inc., Cary, NC, USA).

## 3 RESULTS

### 3.1 Microclimate

**Figure 1:**
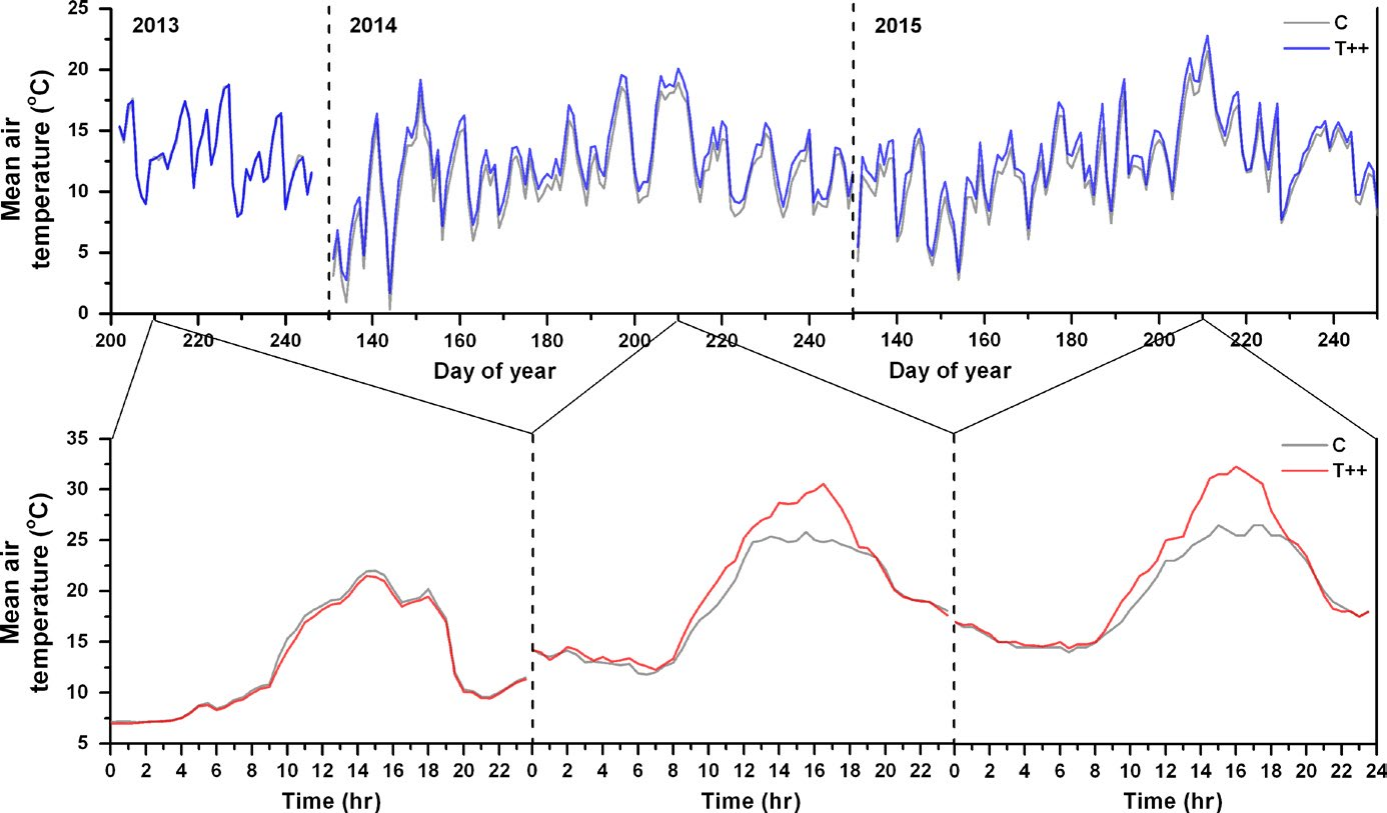
Top: daily mean air temperatures measured at 1.5 m for T++ (blue line) and corresponding control (gray line) treatments in block 1 before chamber construction (2013) and for the duration of the experiment (2014 and 2015). Bottom: hourly values of air temperature for T++ (red line) and corresponding control treatments (gray line) on the 210th day (randomly selected for presentation) of each year (data of the T+ treatment were shown in Supporting Information Figure S2)

Monitoring revealed the relative consistency in micrometeorological conditions among control groups of the blocks (Supporting Information Table S1). Ambient conditions (control groups) during the 2015 growing season (from May to September) were warmer and drier than those during 2014. Specifically, temperature was 0.3°C higher, and rainfall was 23 mm lower in 2015 than in 2014. The OTC performances over the course of the experiment are shown in Table 2 and Supporting Information Table S3. Warming efficacy varied clearly during the growing season and non‐growing season (from October to April, Supporting Information Table S3), with the faster accumulation of thermal time over the growing period. The warming treatments elevated the air temperature during the growing season by 0.3°C for T+ and 1.1°C for T++ (averages of two years) compared to control groups, respectively; this sustained heating was achieved primarily by increasing air temperature during the daylight hours (Figure 1, Supporting Information Figure S2 & Supporting Information Table S4). The corresponding increases in cumulative degree‐days with a base threshold >5°C before the end of growing season were 115.5 ± 17 and 429.7 ± 58 for T+ and T++ on average, respectively (Supporting Information Table S5). Air relative humidity did not differ significantly between chambers and controls and both warming years (all at *p* > 0.1). Topsoil temperature at 10 cm depth exhibited a non‐significant increase for T++, and a decline for T+ (Table 2), possibly due to partial penetration of chambers by sunlight. Soil volumetric water content (VWC) at shallow layer (10 cm depth) was lower in both warming treatments than in controls, especially under T++ conditions. In contrast, there were no significant changes in soil temperature and VWC at deep layer (40 cm depth) between the chambers and control groups in each block (Table 2).

**Table 2:** The effect of warming on mean air and soil temperature, and soil water contents during the growing season (from May to September)

Treatment	Year	*T*_air_ (^o^C)	*T*_soil_10cm_ (^o^C)	VWC__10cm_ (%)	*T*_soil_40cm_ (^o^C)	VWC__40cm_ (%)
T++	2014	+1.1 (0.21)	+0.3 (0.25)	−14.8 (4.67)	+0.1 (0.05)	−3.9 (0.35)
	2015	+1.2 (0.15)	+0.4 (0.31)	−17.1 (3.89)	+0.2 (0.12)	−4.5 (2.68)
T+	2014	+0.2 (0.15)	−0.1 (0.27)	−5.1 (3.67)	−0.1 (0.22)	−2.9 (0.21)
	2015	+0.3 (0.11)	−0.1 (0.34)	−4.7 (5.68)	0 (0.30)	1.2 (3.25)

Notes

Shown are mean values with standard deviation in parentheses.

*T*_air_: mean air temperature at 1.5 m above ground; *T*_soil_10cm_: mean soil temperature at 10 cm soil depth; *T*_soil_40cm_: mean soil temperature at 40 cm soil depth; VWC__10cm_: soil volumetric water content at 10 cm soil depth; VWC__40cm_: soil volumetric water content at 40 cm soil depth.

### 3.2 Phenological responses

**Figure 2:**
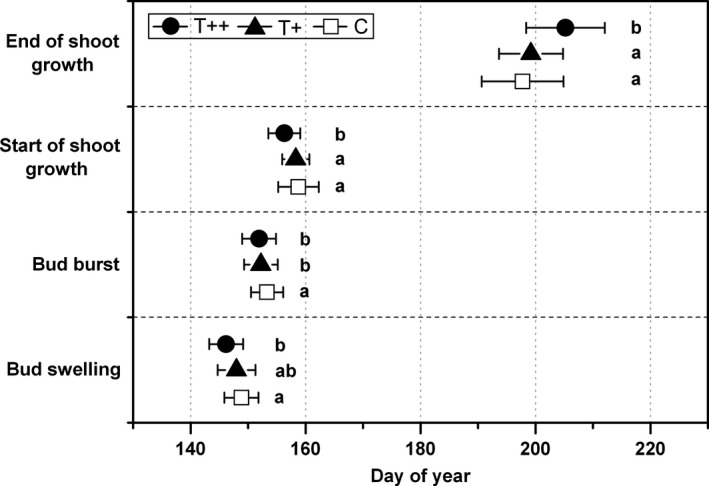
Phenological responses (means ± *SE*) to warming. Values are pooled for both years for all saplings (old and young). One‐way ANOVA was used to detect differences in phenological sensitivity among treatments

Figure 2 showed the phenological responses of all saplings (including old and young) for both years (2014 and 2015) to experimental warming. ANOVA results indicated that temperature increase generally altered phenological timing of *P. crassifolia* saplings, and the dates of bud burst and shoot growth also differed statistically under the different warming regimes. In the T++ chambers, bud swelling occurred approximately 2.9 days earlier (*p = *0.03), the average onset of shoot growth advanced by 2.7 days (*p = *0.04), and cessation of growth tended to be delayed for up to 7.4 days (*p = *0.01) compared to control. In contrast, spruce saplings under T+ conditions exhibited almost no change in the dates of bud burst or cessation of shoot growth (Table 3), probably because the degree of warming did not extend beyond the year‐to‐year variability (the departure from the normal growing season temperature of the past 15 years ranged from −0.3 to 0.65°C).

**Table 3:** Phenological sensitivity of two populations (old and young) to experimental warming

Treatment	Life stage	Bud swelling (days)	Bud burst (days)	Start of shoot growth (days)	End of shoot growth (days)
T++	Old sapling	−4.7 (1.8)**	−2.5 (0.5)*	−2.6 (1.5)*	8.7 (1.9)***
	Young sapling	−1.1 (1.0)	−1.0 (1.3)	−2.4 (0.9)*	3.8 (0.5)**
T+	Old sapling	0.5 (2.7)	−0.3 (0.7)	−0.7 (1.8)	1.8 (1.2)
	Young sapling	−1.5 (2.8)	−0.9 (0.6)	−0.1 (1.4)	1.6 (0.8)

Notes

Values were pooled for both years and calculated as ${\text{(Phenology}}_{\text{warming}}-{\text{Phenology}}_{\bar{\text{control}}})$ where ${\text{Phenology}}_{\bar{\text{control}}}$ represented the averaged measurements of controlled groups. Standard deviations are given in parentheses. The paired *t* tests were used to evaluate the treatment effects.

* *p* < 0.05.

** *p* < 0.01.

*** *p* < 0.001.

Spruce saplings exhibited similar phenological patterns of earlier bud burst and extension of the shoot‐growing season in response to warming in both age‐groups in the combined warming treatments (Table 3). However, there was significant difference in the degree of change with respect to age. Timing of several phenological events in old saplings tended to be highly responsive to the increased temperature; this was especially true for bud swelling and cessation of shoot growth under T++ treatment. The age x treatment interaction was significant for all phenological events except for the timing of onset of shoot growth (Table 4). For example, the mean end‐date of growth in the T++ chambers was an average of 5 days later for old than for young saplings and later for both ages than in T+ chambers and control groups.

**Table 4:** ANOVA results for timing of bud burst and shoot growth

Factor	Bud swelling	Bud burst	Start of shoot growth	End of Shoot growth
	*F*	*F*	*F*	*F*
Age (A)	7.27_(1,468)_ **	17.97_(1,468)_ ***	10.65_(1,468)_ **	51.07_(1,468)_ ***
Treatment (T)	10.68_(2,468)_ ***	4.26_(2,468)_ *	9.53_(2,468)_ ***	28.89_(2,468)_ ***
Year (Y)	2.98_(1,468)_	0.69_(1,468)_	3.01_(1,468)_	195.49_(1,468)_ ***
A × T	3.31_(2,468)_ *	4.32_(2,468)_ *	2.68_(2,468)_	10.17_(2,468)_ ***
A × Y	0.29_(1,468)_	1.23_(1,468)_	2.94_(1,468)_	0.66_(1,468)_
T × Y	0.99_(2,468)_	1.44_(2,468)_	1.66_(2,468)_	9.45_(2,468)_ ***
A × T × Y	9.01_(2,468)_ ***	7.45_(2,468)_ **	7.41_(2,468)_ **	0.36_(2,468)_

Notes

Shown are *F*‐statistics, with degrees of freedom in parentheses. Multiplication between factors represents their interactions.

* *p* < 0.05.

** *p* < 0.01.

*** *p* < 0.001.

### 3.3 Growth response

**Figure 3:**
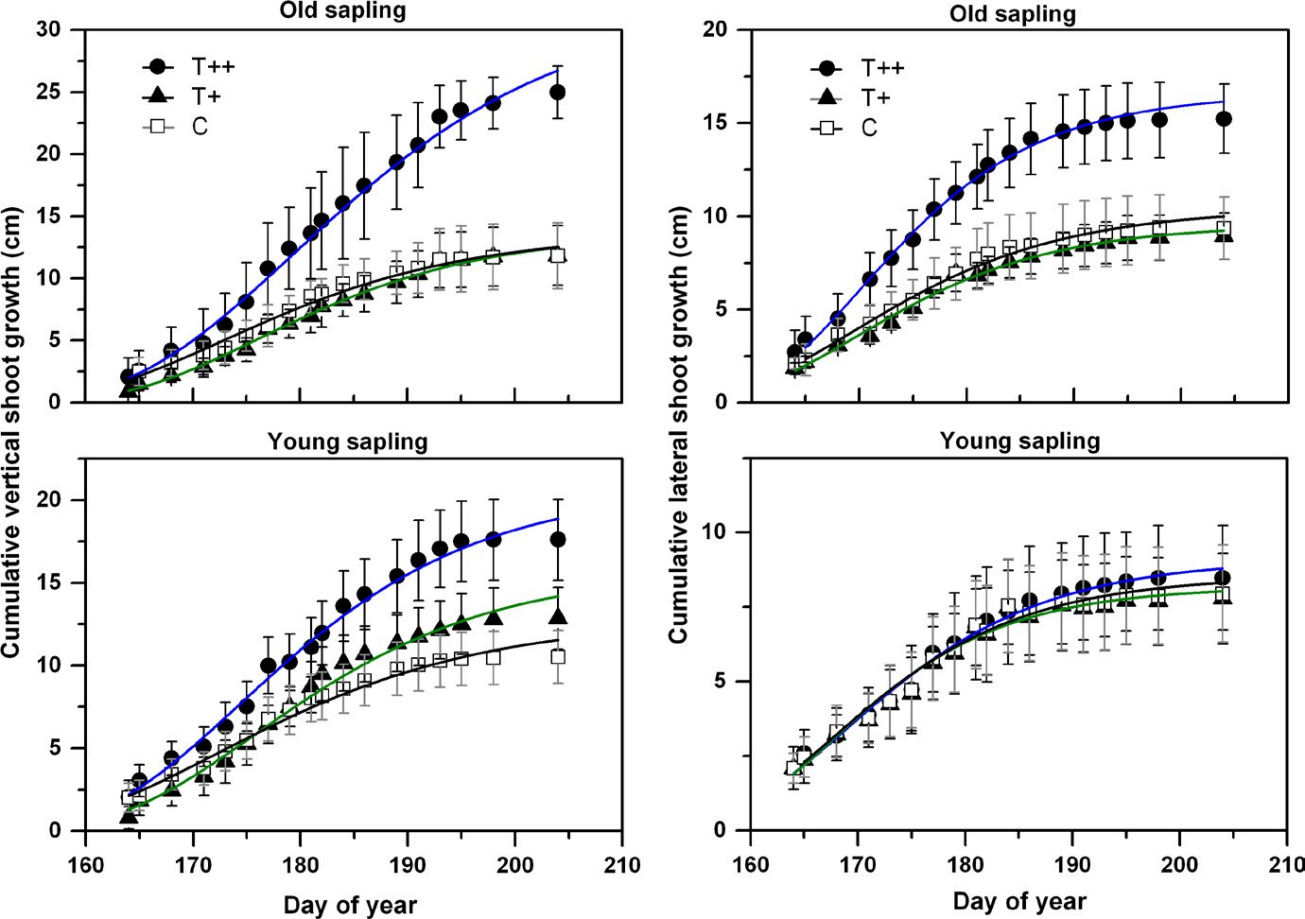
Vertical and lateral growth (means ± *SE*) for old and young saplings within treatments. Data were grouped for both years for warmed and control treatments. Primary growth was fitted with the Gompertz function

**Figure 4:**
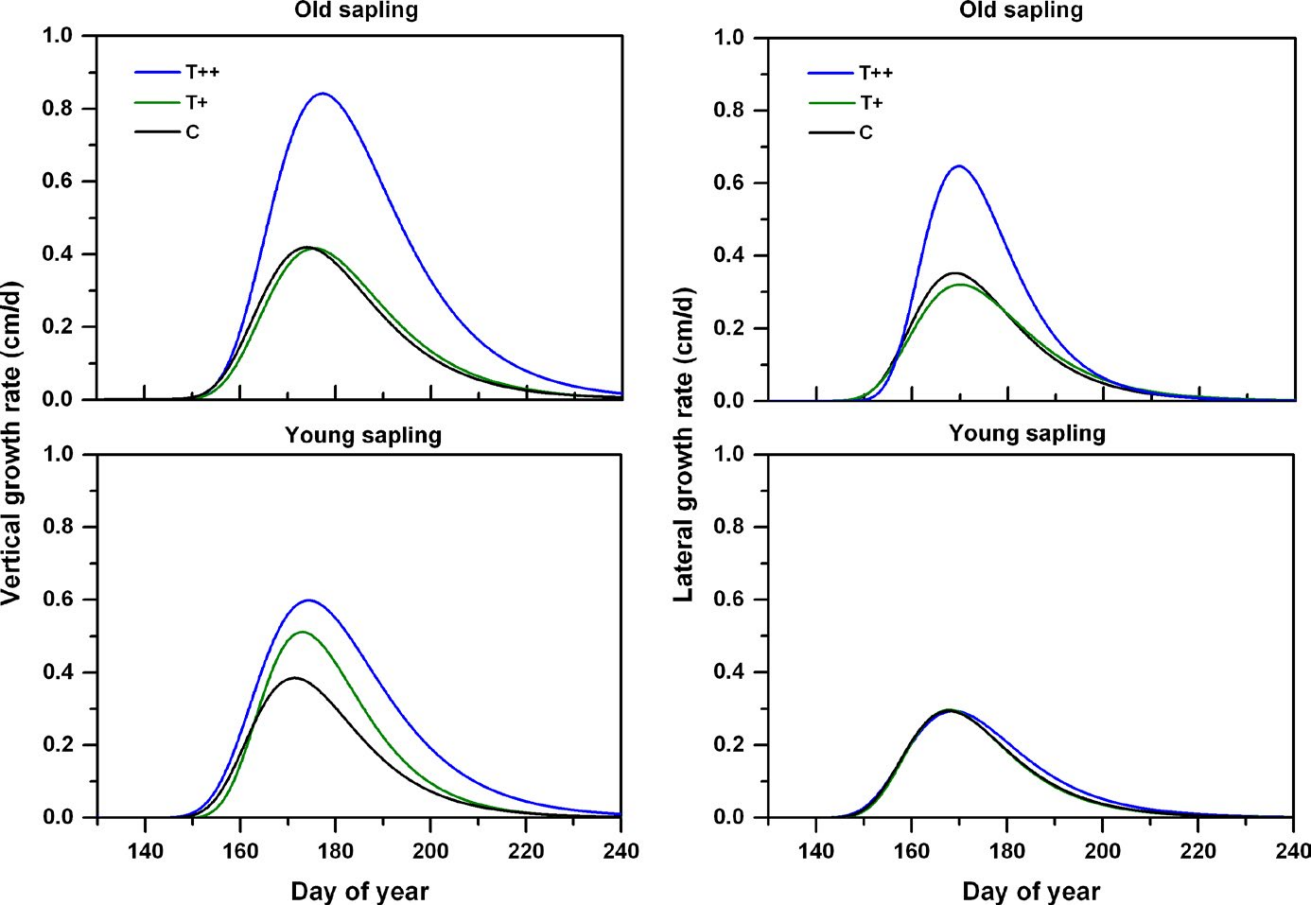
Average growth rates of vertical and lateral growth for two sapling populations in different treatments

The Gompertz function fitted primary growth (including the vertical leaders and lateral branches) well regardless of sapling age, with an adjusted *R* ^2^ of 0.98 (Figure 3). The data exhibited a normally distributed curve of shoot growth rates during the growing season (Figure 4), in which the peak values occurred around mid‐June for lateral shoots to late‐June for vertical shoots. Growth rate was markedly accelerated by warming in T++ and was comparable in T+ to that in the control treatment. For vertical leaders and lateral shoots, average growth rate was inconsistent among years, with the interaction of warming effects. For example, the relative increment of growth rate induced by warming was greater by 18% in 2015 than 2014. Final cumulative leader shoot lengths for old saplings averaged 37% and 10% longer than for young saplings in T++ and C treatments, respectively, with a significant age × treatment interaction. In general, growth of vertical leaders in the control was greater than that of lateral shoots, but this difference was not significant (*p* = 0.26). However, differences between vertical leaders and lateral shoots became greater with an increase in warming level, as indicated by the 50% and the twofold increase in average growth rates in T+ and T++ treatments, respectively (Figure 4). The significant interaction between age and treatment indicated a greater rate of growth for old saplings in T++ than in other treatments (Table 5).

**Table 5:** ANOVA results for growth rates for vertical and lateral growth

	Maximum growth rate		Average growth rate	
	Vertical growth	Lateral growth	Vertical growth	Lateral growth
	*F*	*F*	*F*	*F*
Age (A)	40.22_(1,468)_ ***	48.18_(1,468)_ ***	64.24_(1,468)_ ***	60.47_(1,468)_ ***
Treatment (T)	33.93_(2,468)_ ***	14.35_(2,468)_ ***	67.07_(2,468)_ ***	19.56_(2,468)_ ***
Year (Y)	1.72_(1,468)_	0.93_(1,468)_	20.13_(1,468)_ ***	11.36_(1,468)_ **
A × T	4.44_(2,468)_ *	4.78_(2,468)_ *	13.40_(2,468)_ ***	15.67_(2,468)_ ***
A × Y	0.32_(1,468)_	2.35_(1,468)_	2.41_(1,468)_	1.25_(1,468)_
T × Y	12.76_(2,468)_ ***	9.29_(2,468)_ ***	18.92_(2,468)_ ***	10.18_(2,468)_ ***
A × T × Y	1.93_(2,468)_	2.47_(2,468)_	0.46_(2,468)_	0.56_(2,468)_

Notes

Shown are *F*‐statistics, with degrees of freedom in parentheses. Multiplication between factors represents their interactions.

* *p* < 0.05.

** *p* < 0.01.

*** *p* < 0.001.

**Figure 5:**
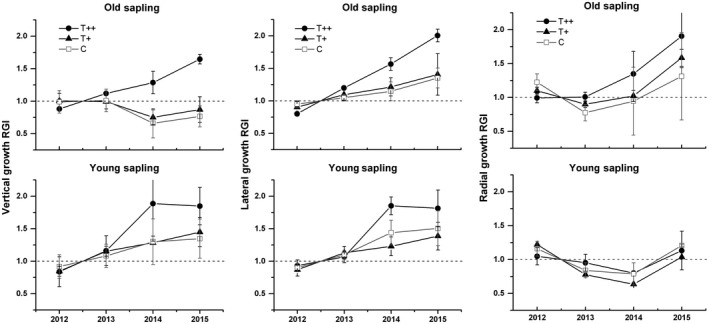
The relative growth index (RGI; means ± *SE*) of primary and radial growth for two sapling populations and treatments in each monitoring year

**Figure 6:**
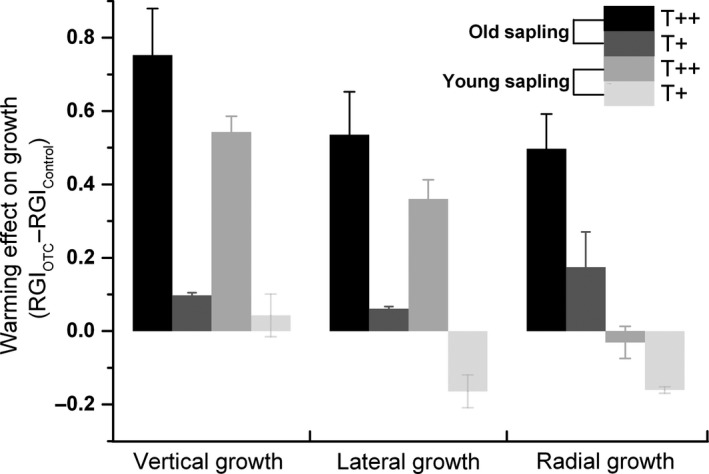
Shoot growth sensitivity of two populations (old and young) to experimental warming. Values were pooled for both years and calculated as ${\text{(RGI}}_{\text{warming}}-{\text{RGI}}_{\bar{\text{control}}})$ where ${\text{RGI}}_{\bar{\text{control}}}$ represented the averaged measurements of controlled groups

The averaged RGI generally increased over the four‐year measuring period for branches, but was inconsistent for stem radial growth (Figure 5); this was likely a result of the interannual variability in resource allocation between height and diameter increment. Saplings in the T++ chambers tended to exhibit a greater relative growth rate than those in T+ chambers and control groups. This divergent response was significant for treatments and years (Table 6). Yearly variability in RGI of primary growth was most likely driven by annual changes of the cessation date of shoot growth as well as the dynamics of average shoot growth rate. For old saplings, the extent to which warming accelerated shoot growth also varied by branch position, with vertical leaders exhibiting a greater response than lateral shoots (Figure 6); these differences were marginally significant at *p* = 0.05 for T+, and *p* = 0.07 for T++ treatments. This was also the case for shoot growth in young saplings. Unlike branch elongation, radial growth exhibited more year‐to‐year variability (Table 6), indicating that the annual variability in climate conditions affected cambial activity. Generally, old saplings exhibited more evident responses to experimental warming than young saplings for all primary growth variables measured in this study (Figure 6).

**Table 6:** ANOVA results for the relative increments for primary and radial growth

	Vertical growth	Lateral growth	Radial growth
	*F*	*F*	*F*
Age (A)	111.81_(1,468)_ ***	5.74_(1,468)_ **	20.75_(1,228)_ ***
Treatment (T)	69.11_(2,468)_ ***	66.68_(2,468)_ ***	2.77_(2,228)_ *
Year (Y)	6.80_(1,468)_ **	9.38_(1,468)_ **	21.71_(1,228)_ ***
A × T	1.68_(2,468)_	3.61_(2,468)_ *	2.80_(2,228)_ *
A × Y	2.01_(1,468)_	8.54_(1,468)_ ***	0.51_(1,228)_
T × Y	0.02_(2,468)_	0.22_(2,468)_	0.04_(2,228)_
A × T × Y	1.83_(2,468)_	2.78_(2,468)_ *	0.25_(2,228)_

Notes

Shown are *F*‐statistics, with degrees of freedom in parentheses. Multiplication between factors represents their interactions.

* *p* < 0.05.

** *p* < 0.01.

*** *p* < 0.001.

## 4 DISCUSSION

Studies of the responses of natural ecosystems worldwide to experimental warming are important for our ability to predict ecological changes at different spatial scales (Aronson & McNulty, 2009). Passive OTCs are extensively used as the simplest and most cost‐effective systems to achieve warming effects (Bronson et al., 2009; De Frenne et al., 2010; Fu et al., 2013). In this study, we improved the experimental design that was normally used in prior studies, and altered the intensity of thermal convection inside the OTCs by adjusting the height of the chambers. By monitoring environmental variables, we confirmed that this method resulted in distinct warming levels inside the chambers without affecting other parameters except for wind and topsoil moisture. However, the warming was achieved primarily by increasing air temperature during the daylight hours, with no obvious changing of nighttime air temperatures. This nonuniformity of sustained heating in daily temporal distribution is the primary shortcoming of passive OTCs, as observed in other studies (Danby & Hik, 2007; Xu et al., 2012).

### 4.1 Warming effects on sapling phenology and growth

Experimental warming of approximately 1.0°C generally advanced the average timing of spring phenology and delayed the cessation dates of shoot growth in Qinghai spruce saplings growing at the low‐elevation treeline. The observed shifts in spring phenology in response to warming confirm the results of previous studies that indicated trends toward an earlier start of vegetative growth with an increase in temperature (Bronson et al., 2009; Dorji et al., 2013; Gunderson et al., 2012; Kaye & Wagner, 2014; Morin, Roy, Sonié, & Chuine, 2010; Prieto et al., 2009). However, the magnitude of plant response to artificial warming is prone to mismatch, generally less than, those of natural variation based on long‐term records. For example, our previous study of more than 10 years of records of remote‐sensing‐derived phenology revealed for *Picea crassifolia* at the same site a mean advance of the growing season of 5.5 days/^o^C, a 40% higher than the current experimental results (Du et al., 2014). In comparison with long‐term observational datasets, Wolkovich et al. (2012) have cautioned about the potential under‐prediction of plant phenological responses to experimental warming, and suggested that the discrepancy between experiments and observations may result from the dampening effect of lower irradiance and drier soils in the experiments, or the interactions of multiple drivers (e.g., snowmelt and photoperiod) in the observations. Additionally, when undergoing the intense warming (e.g., the higher level of experimental warming in our study), saplings may produce a natural resistance to the changing environment (Chuine, 2010). This mechanism allows individuals, in some measure, to maintain the pervious inertial growth performance, possibly leading to a decline of temperature sensitivity (Signarbieux et al., 2017). With the increase of experimental warming duration, such legacy effect became weak, as was indicated by the fact that the relative increment of growth rate induced by warming was greater in 2015 than 2014.

Spruce saplings under the greater warming scenario in our study responded with increased shoot‐growth rate and relative growth for all branch positions and both sapling ages (Figures 4 and 5). Shoot elongation mainly benefited from the increase in a prolonged photosynthetic season due to earlier growing onset and later offset under warmer conditions. In addition, this growth stimulation may also be related to the temperature‐induced increase in soil N availability. A steady decline of the C:N ratios was observed with decreasing elevation (and a temperature increase) at soil depths 0–50 cm at this site; this is known to benefit aboveground growth of *P. crassifolia* (Chen et al., 2016). Other studies also reported that warming tended to increase soil N‐mineralization (Aerts, Cornelissen, & Dorrepaal, 2006; Natali, Schuur, & Rubin, 2012; Xu et al., 2012) and thus to change resource allocation among tissues (Danby & Hik, 2007). In this study, for example, we detected a measurable difference in the final shoot lengths between successive branch whorls, with vertical shoots growing more than lateral ones under warming (Figure 6). Our results suggested that the elevated temperature was likely to accelerate forest development of Qinghai spruce from juvenile sapling stage to maturity. However, unlike primary growth, radial growth exhibited a weaker response to experimental warming (Figure 5); this may be due to an increased dependence on limited soil moisture in late summer and early fall (Fang et al., 2012). The negative impact of sustained low water potentials during the period of cambial activity probably counterbalanced the active contribution from an extended photosynthetic season, thus leading to year‐to‐year variability in tree‐ring widths (Barber, Juday, & Finney, 2000).

Interestingly, the timing of bud burst and the final shoot length did not differ significantly between T+ and the control treatment, even in 2014, when rainfall was relatively abundant. The general lack of sensitivity of Qinghai spruce to low‐level warming was contrary to our expectation that a small change in temperature would be sufficient for a growth response. These results may reflect the observation that temperature was not the overriding determinant of phenology and growth when the increase was less than the range of annual variability (Marchin, Salk, Hoffmann, & Dunn, 2015). This finding further implied an adaptation mechanism of sapling growth in a changing climate. Long‐term adaptation to stressful environment may involve an increase in ecosystem stability and resilience (Pau et al., 2011). Therefore, a critical step in the improvement of future predictions of phenological responses to warming is to focus on warming scenarios that extend the historical range in temperature.

### 4.2 Age‐mediation of sapling responses to warming

As expected, tree age mediated the response of Qinghai spruce to experimental warming. Contrary to our hypothesis, however, old, rather than young, saplings were more sensitive to warming, which disagreed with previous studies of deciduous tree species (Augspurger & Bartlett, 2003; Li et al., 2013). For deciduous woody species, earlier flushing in understory seedlings has been interpreted as an opportunistic strategy to enhance light gains and exploit greater light availability for growth before canopy closure (Vitasse, 2013). In contrast, light response of shade‐tolerant evergreen confers like Qinghai spruce normally lacks its sensitivity for sapling growth, because light transmission in canopy layers rarely exhibits seasonal variation throughout the year (Du et al., 2014). Although the differences in photosynthetic capacity, light response, morphology, and anatomy between deciduous and evergreen woody species have been investigated (Antúnez, Retamosa, & Villar, 2001; Walters & Reich, 1999), few studies monitored temperature sensitivity of evergreen growth across ontogenetic stages to date. A recent study of dragon spruce in the eastern Tibetan Plateau also found a similar age‐effect in mediating tree performance in response to experimental warming, with 8‐year‐old saplings responding more than 2‐year‐old seedlings for bud burst and stem biomass (Xu et al., 2012). Our results partially agree with this study and may indicate an increase of phenotypical plasticity with tree age in response to environmental variability (Cheplick, 1995).

Experimental warming often causes the decrease of soil moisture, thus leading to the increase of water stress (Dorji et al., 2013; Morin et al., 2010). In this study, visible changes were also found for the water availability of the topsoil in high‐level warming treatment. This may partially affect the sapling growth in response to experimental warming, due to the different rooting depths between age classes. However, in view of their final performances (i.e., elongation of growth period and promotion of shoot increment), the decreasing of the topsoil water content had a minor influence on the vegetative phenology and shoot growth of both saplings. At the very least, our results indicated that warming exerted more positive than negative effects on Qinghai spruce. Therefore, other potential mechanisms are probable in accounting for the age‐related performance.

It is noteworthy that OTC experiment significantly produces the asymmetric warming during the day (Figure 1 and Supporting Information Table S3). Since plant photosynthesis in most cases occurs during daytime and shows a great sensitivity to the maximum daily temperature, daytime warming is expected to be beneficial for carbon fixation and energy capture (Peng et al., 2013). It may be especially the case for old sapling growth, because several morphological and physiological indices reflecting the photosynthetic capacity of Qinghai spruce, such as stomatal density, area‐based leaf nitrogen concentration, and net photosynthetic rate, were normally observed to increase with tree age before maturity in the same natural habitats (Zhao, Chen, Ma, Yao, & Liu, 2008). In contrast, plant autotrophic respiration during nights is mainly a process of energy consumption (Turnbull, Murthy, & Griffin, 2002). Leaf carbohydrates synthesized during the daytime were investigated to be consumed quickly during warm nights (Griffin et al., 2002). Accordingly, our results of increased daytime temperature would significantly enhance leaf photosynthesis, whereas lack of obvious nighttime warming would effectively restrict autotrophic respiration, thus resulting in the higher growth rate of old saplings compared with young saplings. In addition, the differences in phenological response of the two age groups may also be related to the low night temperatures at springtime and the associated risk of frost damage. In our site, the daily minimum temperatures during preseason period are often below 5°C, which put a strong constraint on plant bud burst and leaf unfolding (Du et al., 2014; He et al., 2015; Shen et al., 2016). Lack of obvious nighttime warming rendered spruce saplings to be still in high stresses of freezing injury at low temperatures. Due to the lower resistibility to changing conditions compared to old saplings (Zhao et al., 2008), young saplings may exhibit a higher sensitivity to nighttime chilling, and thus, the phenological response to experimental warming is limited.

## 5 CONCLUSIONS

Age‐mediation of temperature sensitivity of tree growth has received little attention, even for deciduous species (Esper et al., 2008; Ruiz‐Benito et al., 2015; Xu et al., 2012). In this study, we explored growth responses of Qinghai spruce to experimental warming in tree saplings at successive stages of development. We found that old saplings had a higher temperature sensitivity than young saplings and that this response subsided when the increase in temperature was lower than the range of annual temperature variability. The differential growth performances between age classes may be due to the differences in adaptive strategy to the asymmetric warming during the day. Although we could not therefore determine such tree performance is consistent during other phases of the life cycle, we did find that age‐mediation of tree‐growth responses to experimental warming in Qinghai spruce was contrary to the common expectations form previous studies of deciduous tree species, presumably indicating a unique mechanism in responding to a changing climate for evergreen trees. Our results suggested that the conclusions obtained from these studies of deciduous tree species may therefore not be necessarily valuable for the inference of climate signal age effects on leaf phenology and shoot growth of evergreen conifers. Further investigations need to be performed on the underlying mechanisms of phenological responses of evergreen species to climate warming.

## CONFLICT OF INTEREST

None declared.

## AUTHOR CONTRIBUTIONS

JD and ZBH conceived and designed the experiments. KL and LFC performed the experiments. JD, XZ, and PFL analyzed the data and wrote the manuscript; all authors provided editorial advice.

## Data Availability

Climate data and warming efficacy: uploaded as online supporting information.
