# Supplementary material for: Age‐mediation of tree‐growth responses to experimental warming in the northeastern Tibetan Plateau
Source: Ecol Evol. 2019 Jan 28;9(4):2242–54. doi: 10.1002/ece3.4920 (PMC6392491; doi:10.1002/ece3.4920)

Supporting information for

**Age-mediation of tree growth responses to experimental warming in the northeastern Tibetan Plateau**

Jun Du^1,2^∣Kai Li^3,4^∣Zhibin He^1,2^*∣Longfei Chen ^1,2^∣Xi Zhu ^1,2,5^∣Pengfei Lin ^1,2,5^

^1^*Linze Inland River Basin Research Station, Chinese Ecosystem Research Network, China*

^2^*Key Laboratory of Ecohydrology of Inland River Basin, Northwest Institute of Eco-Environment and Resources, Chinese Academy of Sciences, Lanzhou 730000, China*

^3^*Key Laboratory of Western China’s Environmental Systems, College of Earth Environmental Sciences, Lanzhou University, Lanzhou 730000, China*

^4^*Department of Agricultural and Applied Economics, Texas Tech University, 2500 Broadway Lubbock,Texas 79409, USA*

^5^*University of Chinese Academy of Sciences, Beijing 100049, China*

Corresponding Author. Tel. 86-931-4967165, *E-mail addresses*: [hzbmail@lzb.ac.cn](mailto:hzbmail@lzb.ac.cn) (Z.B. He).

Table S1

Comparisons of micrometeorological conditions between control groups of the blocks

|  | 2013 (August) ^a^ | | |  | 2014_growing season (from May to September ) | | |  | 2015_growing season (from May to September ) | | |
| --- | --- | --- | --- | --- | --- | --- | --- | --- | --- | --- | --- |
|  | *T*_air_ (^o^C) | *T*_soil_ (^o^C) | VWC (m^3^/m^3^) |  | *T*_air_ (^o^C) | *T*_soil_ (^o^C) | VWC (m^3^/m^3^) |  | *T*_air_ (^o^C) | *T*_soil_ (^o^C) | VWC (m^3^/m^3^) |
| Block 1 | 14.14 | 11.33 | 0.11 |  | 10.37 | 7.74 | 0.10 |  | 10.73 | 7.47 | 0.15 |
| Block 2 | 14.18 | 10.64 | 0.09 |  | 10.48 | 7.09 | 0.08 |  | 10.74 | 6.88 | 0.13 |
| Block 3 | 14.24 | 10.96 | 0.09 |  | 10.57 | 7.71 | 0.09 |  | 10.85 | 7.42 | 0.16 |
| Block 4 | 14.21 | 11.81 | 0.11 |  | 10.63 | 8.28 | 0.11 |  | 10.95 | 8.02 | 0.17 |
| *F*-statistics | — | — | — |  | 0.01 | 0.11 | 2.28 |  | 0.01 | 0.11 | 5.11 |
| *P*-value ^b^ | — | — | — |  | 1.00 | 0.95 | 0.12 |  | 1.00 | 0.95 | **0.01** |

*T*_air_: mean air temperature at 1.5 m above ground; *T*_soil_: mean soil temperature at depth of all soil layers; VWC: soil volumetric water content at depth of all soil layers.

^a^ The chambers were constructed in September, 2013, while the meteorological sensors were installed 1 month before chamber construction

^b^ A Bonferroni’s test was used to detect differences in climatic metrics among blocks. Bolded text indicates *P* values < 0.05

Table S2

Block effects in population characteristics and sapling growth in each age-group using a one-way ANOVA test. Difference is significant in *P* values < 0.05.

| Variables | Young sampling | |  | Old sampling | |
| --- | --- | --- | --- | --- | --- |
|  | *F*-statistics | *P*-value |  | *F*-statistics | *P*-value |
| Bud swelling | 0.348 | 0.558 |  | 0.468 | 0.497 |
| Bud burst | 0.546 | 0.464 |  | 1.725 | 0.196 |
| Start of shoot growth | 1.142 | 0.291 |  | 1.006 | 0.321 |
| End of Shoot growth | 0.015 | 0.981 |  | 0.086 | 0.770 |
| Maximum growth rate | 0.806 | 0.374 |  | 0.842 | 0.364 |
| Average growth rate | 0.003 | 0.954 |  | 0.005 | 0.945 |
| Vertical growth increment | 0.317 | 0.576 |  | 0.015 | 0.903 |
| Lateral growth increment | 0.381 | 0.540 |  | 0.411 | 0.525 |
| Radial growth increment | 1.048 | 0.317 |  | 0.566 | 0.460 |

Table S3

Warming efficacy during non-growing season (from October to April).

| Treatment | Year | *T*_air_ (^o^C) | T_soil_(^o^C) | VWC(%) |
| --- | --- | --- | --- | --- |
| T++ | 2014 | +0.2 (0.13) | +0.15 (0.23) | -6.4 (5.64) |
|  | 2015 | +0.1 (0.38) | +0.23 (0.27) | +0.7 (6.12) |
| T+ | 2014 | -0.2 (0.27) | -0.04 (0.34) | +4.9 (3.68) |
|  | 2015 | -0.2 (0.26) | -0.35 (0.31) | +3.4 (3.17) |

*T*_air_: mean air temperature at 1.5 m above ground; *T*_soil_: mean soil temperature at depth of all soil layers; VWC: soil volumetric water content at depth of all soil layers. Shown are mean values with standard deviation in parentheses.

Table S4

Asymmetric warming during the daylight hours (6:00 am - 18:00 pm) and the nighttime (18:00 pm - 6:00 am)

| Treatment | Year | Daytime (6:00 am - 18:00 pm) | | |  | Nighttime (18:00 pm - 6:00 am) | | |
| --- | --- | --- | --- | --- | --- | --- | --- | --- |
|  |  | *T*_air_ (^o^C) | T_soil_(^o^C) | VWC(%) |  | *T*_air_ (^o^C) | T_soil_(^o^C) | VWC(%) |
| T++ | 2014 | +1.6 (0.16) | +0.31(0.13) | -12.8 (5.51) |  | +0.4 (0.22) | +0.17 (0.18) | -4.7 (4.73) |
|  | 2015 | +1.9 (0.14) | +0.58(0.22) | -17.8 (7.81) |  | +0.2 (0.26) | +0.21 (0.35) | -3.0 (1.69) |
| T+ | 2014 | +0.7 (0.26) | -0.18 (0.29) | -9.2 (3.27) |  | -0.2 (0.31) | -0.07 (0.13) | +1.4 (6.67) |
|  | 2015 | +0.5 (0.13) | -0.26 (0.35) | -5.6 (6.42) |  | +0.1 (0.08) | +0.12 (0.23) | +0.9 (4.99) |

*T*_air_: mean air temperature at 1.5 m above ground; *T*_soil_: mean soil temperature at depth of all soil layers; VWC: soil volumetric water content at depth of all soil layers. Shown are mean values with standard deviation in parentheses.

Table S5

Cumulative growing degree days calculated for control and warming treatments in 2014 and 2015.

| Treatment | 2014 (from Julian day 1 to September) | | |  | 2015 (from Julian day 1 to September) | | |
| --- | --- | --- | --- | --- | --- | --- | --- |
|  | *OTC* | *Control* | Difference |  | *OTC* | *Control* | Difference |
| T++ | 1463.1 (24.1) | 1070.5 (12.6) | 392.6 |  | 1630.6 (30.1) | 1163.8 (13.4) | 466.8 |
| T+ | 1161.3 (18.3) | 1055.2 (16.4) | 106.1 |  | 1292.5 (17.6) | 1167.6 (10.6) | 124.9 |

Shown are mean values with standard deviation in parentheses.

**Figure S1**. Experimental layout of the two-factorial randomized block design in the field. Two levels of warming are denoted by “T++” and “T+”, with “C” for control groups. Old saplings are in block 1 and 3; young saplings in block 2 and 4 (for details, see Table 1).


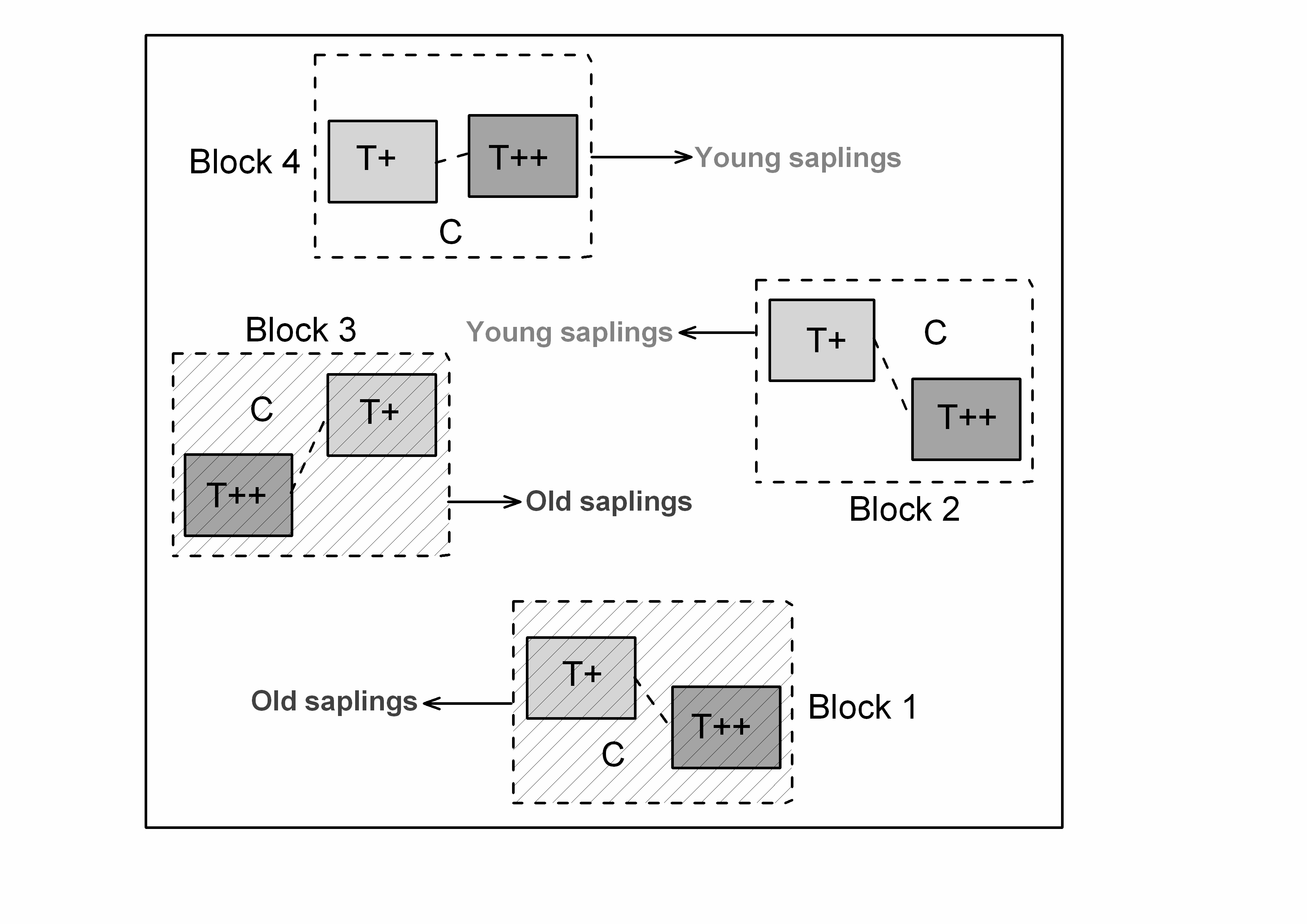


**Figure S2**. Top: daily mean air temperatures measured at 1.5 m for T+ and corresponding control treatments in block 1 before chamber construction (2013) and for the duration of the experiment (2014 and 2015). Bottom: hourly values of air temperature for T+ and corresponding control treatments on the 210th day (randomly selected for presentation) of each year.


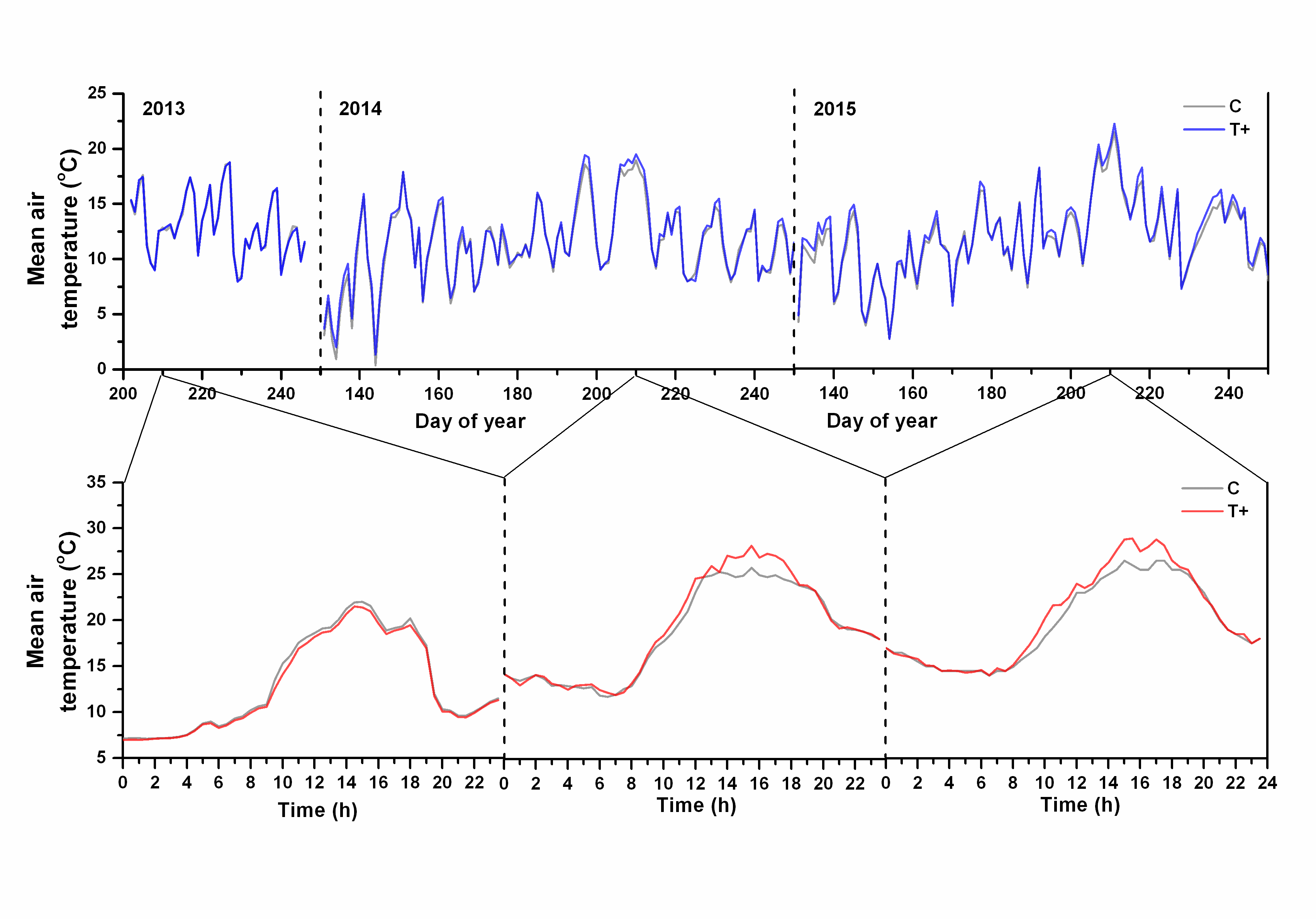

Supplement: Supplementary file 3 [file ECE3-9-2242-s003.doc]
